# Nicotine-Induced Apoptosis in Human Renal Proximal Tubular Epithelial Cells

**DOI:** 10.1371/journal.pone.0152591

**Published:** 2016-03-30

**Authors:** Chang Seong Kim, Joon Seok Choi, Soo Yeon Joo, Eun Hui Bae, Seong Kwon Ma, JongUn Lee, Soo Wan Kim

**Affiliations:** 1 Department of Internal Medicine, Chonnam National University Medical School, Gwangju, Korea; 2 Department of Physiology, Chonnam National University Medical School, Gwangju, Korea; University of Pecs Medical School, HUNGARY

## Abstract

**Background:**

Nicotine is, to a large extent, responsible for smoking-mediated renal dysfunction. This study investigated nicotine’s effects on renal tubular epithelial cell apoptosis *in vitro* and it explored the mechanisms underlying its effects.

**Methods:**

Human proximal tubular epithelial (HK-2) cells were treated with nicotine. Cell viability was examined by using the WST-1 assay. Intracellular levels of reactive oxygen species (ROS) and the expression of mitogen-activated protein kinase (MAPK) and nuclear factor-κB (NF-κB) proteins were determined. The messenger ribonucleic acid and the protein expression associated with the nicotine acetylcholine receptors (nAChRs) in HK-2 cells was examined, and apoptosis was detected using flow cytometry, cell cycle analysis, and immunoblot analysis.

**Results:**

The HK-2 cells were endowed with nAChRs. Nicotine treatment reduced cell viability dose dependently, increased ROS levels, and increased extracellular signal-regulated kinase (ERK), c-Jun N-terminal kinase (JNK), and p38 MAPK expression. Nicotine increased NF-κB activation, which was attenuated by N-acetyl-L-cysteine, and ERK and JNK inhibitors, but was not affected by a p38 MAPK inhibitor. Nicotine increased the Bax/Bcl-2 ratio, which was attenuated by N-acetyl-L-cysteine, the NF-κB inhibitor, Bay 11–7082, and hexamethonium, a non-specific nAChR blocker. Flow cytometry revealed nicotine-induced G2/M phase arrest. While nicotine treatment increased the expression of phosphorylated cdc2 and histone H3, a marker of G2/M phase arrest, hexamethonium and Bay 11–7082 pretreatment reduced their expression.

**Conclusions:**

Nicotine caused apoptosis in HK-2 cells by inducing ROS generation that activated the NF-κB signaling pathway via the MAPK pathway and it arrested the cell cycle at the G2/M phase. Nicotine-induced apoptosis in HK-2 cells involves the nAChRs.

## Introduction

Cigarette smoking is the leading cause of preventable death in the industrialized world, and it is far ahead of other causes of preventable death, including alcohol, drug abuse, and motor vehicle accidents [1]. In addition to its pathologic role in the development of cardiovascular disease, cancer, and chronic obstructive pulmonary disease, the findings from recent epidemiologic studies suggest that cigarette smoking is an independent risk factor for the development and progression of kidney disease [2–5].

Although the findings from recent experimental studies have shown that nicotine promotes mesangial cell proliferation and hypertrophy via non-neuronal nicotinic acetylcholine receptors (nAChRs) in rats with 5/6 nephrectomies [6], the mechanism by which cigarette smoking worsens renal function has not been clearly elucidated. However, nicotine seems to play an important role in smoking-mediated renal dysfunction [6–8]. Nicotine is a major component of cigarette smoke, and is, to a large extent, responsible for the addictive effects of cigarette smoking [9]. Nicotine may deregulate essential biological process, including angiogenesis, apoptosis, and cell-mediated immunity, by binding to the nicotine acetylcholine receptors [10], which are inotropic receptors that function as agonist-regulated calcium channels and are expressed by neuronal as well as non-neuronal cells, including the endothelial cells, vascular smooth muscle cells, and tubular epithelial cells [11–13]. Apoptosis is the process of programmed cell death, and it plays a central role in the physiological processes underlying kidney growth and remodeling and in various renal diseases [14–16]. Notably, proximal tubular epithelial cells are highly susceptible to apoptosis, and injury at this site contributes to renal failure [17, 18]. Nicotine has been observed at high concentrations in the blood and kidneys of chronic smokers [19]; therefore, the renal tubular cells are exposed to nicotine via glomerular filtration and the tubular secretion of nicotine, which may result in direct tubular toxicity [7]. Given the widely recognized deleterious effect of nicotine on the progression of kidney disease, it is conceivable that nicotine may promote tubular injury in human renal tubular epithelial (HK-2) cells. In the present study, we aimed to determine whether HK-2 cells possess nAChRs and whether nicotine promotes apoptosis in HK-2 cells. Furthermore, we investigated the molecular mechanisms underlying apoptosis and whether cell cycle arrest is involved in apoptosis in HK-2 cells treated with nicotine. Therefore, our study may help to determine the pathophysiology of nicotine-mediated renal dysfunction.

## Materials and Methods

### Primary antibodies

The primary antibodies used were anti-rabbit antibodies against extracellular signal-regulated kinase (ERK) (9102), phosphorylated ERK (p-ERK) (9101), c-Jun N-terminal kinase (JNK) (9258), phosphorylated c-Jun N-terminal kinase (p-JNK) (9251), p38 mitogen-activated protein kinase (MAPK) (8690), phosphorylated p38 MAPK (p-p38 MAPK) (4631), Bax (2772), Bcl-2 (2870), the nuclear factor-κB (NF-κB) p65 subunit (3034), cyclin B1 (4138), phosphorylated cdc2 (Tyr 15) (9111), phosphorylated histone H3 (Ser 10) (3377), and histone H3 (9715), all of which were obtained from Cell Signaling Technology, Inc. (Beverly, MA), and anti-rabbit antibodies against nAChR α3 (NBP1-18793), nAChR α5 (NBP1-69122), and nAChR β1 (ANC-001), which were obtained from Novus Biochemicals (Littleton, CO) and Alomone Labs (Jerusalem, Israel). Anti-rabbit antibodies against IκBα (SC-371) and β-actin (A3854) were obtained from Santa Cruz Biotechnology, Inc. (Dallas, TX) and Sigma-Aldrich Co. (St. Louis, MO), respectively.

### Cell culture and reagents

The HK-2 cells (American Type Culture Collection, Manassas, VA), were cultured in Dulbecco’s Modified Eagle’s Medium/F-12 medium (DMEM-F12; Sigma-Aldrich Co., St. Louis, MO), as previously described [20]. The cells were treated with nicotine (N3876; Sigma-Aldrich Co., St. Louis, MO). PD 98059, an ERK inhibitor (513000), SP 600125, a specific JNK inhibitor (420119), and SB 203580, a p38 MAPK inhibitor (559387), were obtained from Calbiochem (San Diego, CA), N-acetyl-L-cysteine (NAC) (A7250) and hexamethonium chloride (H2138) were obtained from Sigma-Aldrich Co. (St. Louis, MO), and Bay 11–7082, an NF-κB inhibitor (Cay-10010266) was obtained from BioMol GmbH (Hamburg, Germany).

### Determination of reactive oxygen species generation

Intracellular reactive oxygen species (ROS) generation was measured with the fluoroprobe, 2′,7′-dichlorodihydrofluorescein diacetate (DCF-DA) (Molecular Probes, Wilmington, DE). The cells were incubated in fresh medium containing 0, 50, 100, 200, or 400 μM of nicotine for 24 h. At the end of the treatment period, the cells were incubated with 5 μM DCF-DA for 30 min at 37°C, as previously described [21]. The images were obtained using a fluorescence microscope (Nikon Corporation, Tokyo, Japan).

### Cell viability assay

To examine the effect of nicotine on cell viability, the cells (1 × 10^4^ cells per well) were seeded into 96-well culture plates and they were incubated for 24 h with or without nicotine (0, 50, 100, 200, or 400 μM). Cell viability was determined using the EZ-CyTox (tetrazolium salt, WST-1) cell viability assay kit (Daeil Lab Inc, Seoul, Korea), as described previously [22]. Cell viability was expressed as a fraction of the surviving cells relative to the untreated control cells.

### Messenger ribonucleic acid expression associated with the nicotine acetylcholine receptors

The messenger ribonucleic acid (mRNA) expression associated with the nAChRs was determined using the reverse transcription-polymerase chain reaction (PCR). Complimentary deoxyribonucleic acid (cDNA) was generated by reverse transcribing 5 μg of total ribonucleic acid (RNA) using an oligo (dT) primer and Superscript Reverse Transcriptase II (Invitrogen Corp., Carlsbad, CA). The cDNA was quantified using the Smart Cycler II System (Cepheid Inc., Sunnyvale, CA), and SYBR^®^ Green was used for its detection. The PCR was undertaken using the Rotor-Gene^TM^ 3000 Detection System (Corbette Research Pty Ltd, Mortlake, New South Wales, Australia), as follows: 1) 95°C for 5 min; 2) 95°C for 30 s; 3) 72–95°C for 30 s (optimized for each primer pair); and 4) 72°C for 7 min, and this cycle was repeated for an additional 30 cycles. The primer sequences were selected from the unique cytoplasmic domain regions of each nAChR subunit, and they were prepared as described previously [23]. Table 1 lists each nAChR primer. Glyceraldehyde-3-phosphate dehydrogenase was used as the internal control to verify the efficiency of the RNA isolation and the cDNA synthesis. The PCR products were run on a 1.5% agarose gel containing ethidium bromide, and they were visualized under ultraviolet illumination.

**Table 1:** Primer sequences.

**Gene**	**Forward primer (5′–3′)**	**Reverse primer (5′–3′)**	**Product size (base pair)**	**Annealing temperature (°C)**
**α1**	CGT TCT GGT GGC AAA GCT	CCG CTC TCC ATG AAG TT	580/505	62
**α2**	CCG GTG GCT TCT GAT GA	CAG ATC ATT CCA GCT AGG	466	48
**α3**	CTG GTG AAG GTG GAT GAA GT	CTC GCA GCA GTT GTA CTT GA	464	62
**α4**	GGA TGA GAA GAA CCA GAT GA	CTC GTA CTT CCT GGT GTT GT	444	62
**α5**	GAT AAT GCA GAT GGA CGT	TGA TGG TAT GAT CTC TTC	525	55
**α6**	GTG GCC TCT GGA CAA GAC AA	CCT GCA GTT CCA AAT ACA CA	372	58
**α7**	CCT GCA GTT CCA AAT ACA CA	CAG CGT ACA TCG ATG TAG CA	375	51
**β1**	CTA CGA CAG CTC GGA GGT CA	GCA GGT TGA GAA CCA CGA CA	479	62
**β2**	CAA TGG CTC TGA GCT GGT GA	CCA CTA GGT GTG AAG TCG TCC A	453	48
**β3**	TGGAGA GTA CCT GCT GTT CA	CGA GCC TGT TAC TGA CAC TA	439	62
**β4**	GTG AAT GAG CGA GAG CAG AT	GGG ATG ATG AGG TTG ATG GT	524	62

### Western blot analysis

The cells were harvested, washed twice with ice-cold phosphate-buffered saline (PBS), resuspended in lysis buffer, and sonicated briefly. After centrifugation, the supernatants were prepared as protein extracts, and the protein concentrations were measured using a Pierce^®^ BCA Protein Assay Kit (Pierce Biotechnology, Inc., Rockford, IL). Equal concentrations of protein were separated on 9% or 12% sodium dodecyl sulfate polyacrylamide gels, and the proteins were transferred onto nitrocellulose membranes. The blots were blocked with 5% milk in PBS-T for 1 h. The blots were then incubated overnight at 4°C with the primary antibodies, which was followed by incubation with the anti-rabbit or anti-mouse horseradish peroxidase-conjugated antibodies, as described previously [24]. The labeling was visualized using an enhanced chemiluminescence system.

### Nuclear extracts preparation

To prepare the nuclear extracts, the cells were lysed using the NE-PER^®^ nuclear extraction reagent (NER) (Pierce Biotechnology, Rockford, IL), as previous described [20]. Briefly, the HK-2 cells that had been incubated with nicotine were harvested, and they were centrifuged at 14,000 *g* for 2 min. After removing the supernatant, ice-cold cytoplasmic extraction reagent (CER) I (100 mL) was added to the dried cell pellets, and, after incubation on ice for 10 min, ice-cold CER II was added to the tube. The tube was centrifuged at 16,000 *g* for 5 min and the pellet fraction was suspended in 50 mL of ice-cold NER. After centrifuging the tube at 16,000 *g* for 10 min, the supernatant containing the nuclear extract fraction was transferred to a clean tube.

### Cell cycle analysis

To identify the distribution of the cell cycle phases among the cells, HK-2 cells that had been exposed to nicotine at 200 or 400 μM for 16 h were stained using phosphorylating histone H3 and propidium iodide (PI) and the cell cycle detection kit (FCCH025103; FlowCellect™ Bivariate Cell Cycle Kit, Merck Millipore, Darmstadt, Germany), according to the manufacturer’s instructions. The fluorescence of the phosphorylated histone H3- and PI-stained cells was measured using flow cytometry (FACS Calibur™; Becton, Dickinson and Company, San Jose, CA).

### Annexin V/propidium iodide staining assay

HK-2 apoptosis was assessed using the Annexin V-fluorescein isothiocyanate (FITC) Apoptosis Detection Kit (K29100; Koma Biotech, Seoul, Korea) in accordance with the manufacturer’s instructions. After exposure to 200 μM nicotine for 24 h, the HK-2 cells were harvested, washed with pre-cooled PBS, and they were re-suspended in a binding buffer containing FITC-conjugated annexin-V protein and PI. After incubation in the dark for 30 min, the annexin V binding and PI staining were determined using flow cytometry. The percentages of the cells residing in the lower right (apoptotic cells) and upper right (necrotic cells) regions of the annexin V-FITC scatter plots were calculated for the comparisons.

### 4′-6-diamidino-2-phenylindole staining assay

The apoptotic nuclei were detected using 4′-6-diamidino-2-phenylindole (Invitrogen, Seoul, Korea), which is a deoxyribonucleic acid-specific fluorescent dye, as previously described [25].

### Statistical analysis

The data are expressed as the means ± the standard errors of the means. The statistical analyses were performed using the Mann-Whitney *U*-test or the paired Student’s *t*-test, as appropriate. Differences were considered statistically significant when the values of *P* were < 0.05. The statistical analyses were performed using statistical software (GraphPad Software Inc., San Diego, CA).

## Results

### Nicotine acetylcholine receptor expression in HK-2 cells

**Fig 1:**
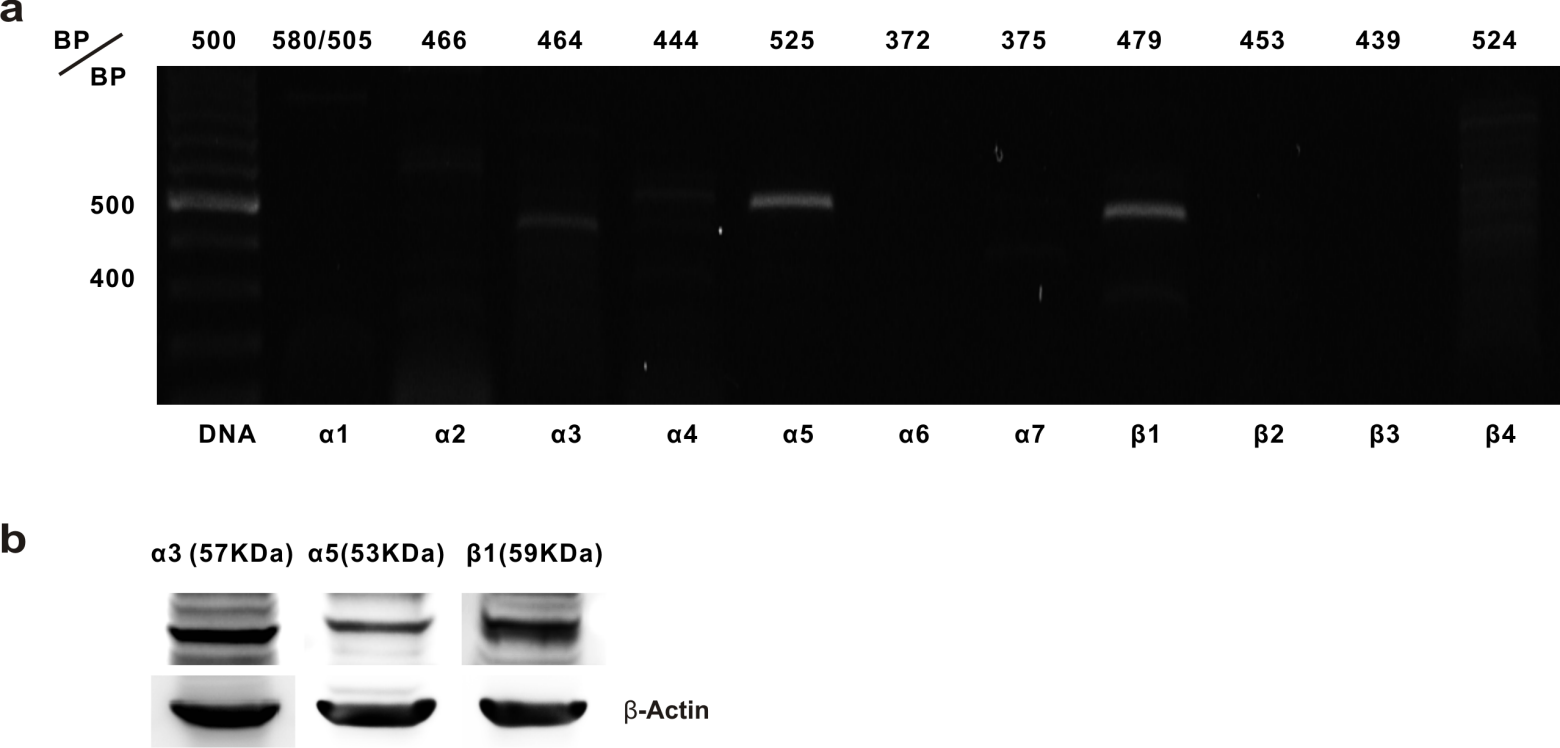
Expression of the nicotine acetylcholine receptors’ (nAChRs) messenger ribonucleic acid (mRNA) in human tubular epithelial cells. **(a)** The mRNA expression associated with the α1–7 and β1–4 nAChR subunits in HK-2 cells was analyzed using the reverse transcription-polymerase chain reaction. (b) Western blot analysis determined the expression of the proteins for the α3, α5, and β1 subunits in HK-2 cells.

We analyzed the HK-2 cells using the reverse transcription-PCR to determine which nAChR subunits, namely, α1–7 or β1–4, were expressed. As shown in Fig 1A, mRNA associated with the α3, α5, and β1 subunits was found in the HK-2 cells. The primers for each subunit yielded products of the expected sizes. However, we could not detect significant levels of mRNA expression associated with the α1, α2, α4, α6, α7, β2, or β3 subunits. Fig 1B shows the expression of the proteins for the α3, α5, and β1 nAChR subunits in HK-2 cells.

### Effects of nicotine on cell viability and reactive oxygen species generation

**Fig 2:**
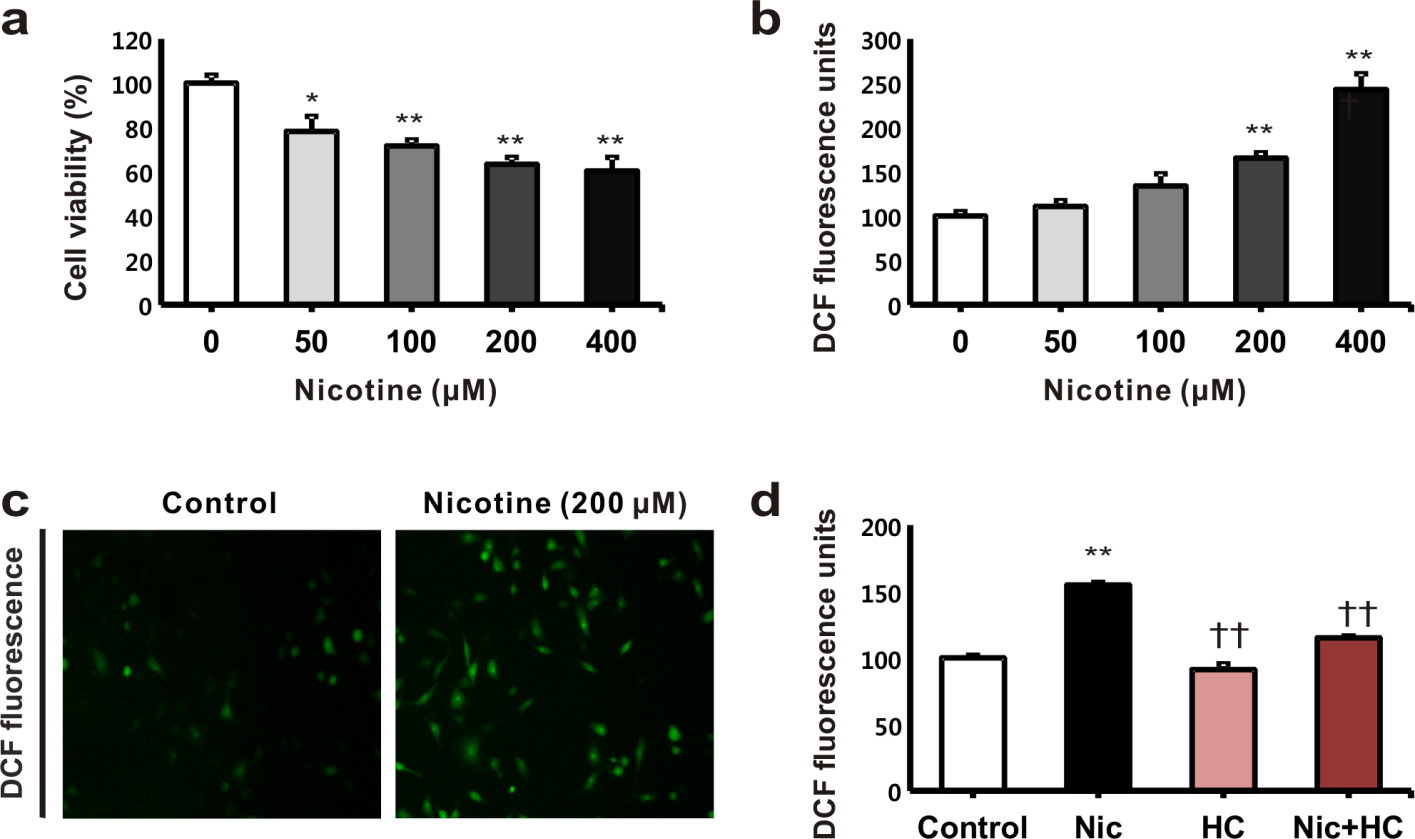
The effects of nicotine on cell viability and reactive oxygen species (ROS) generation in human tubular epithelial cells. (a) The HK-2 cells were treated with nicotine at different concentrations, namely, 0, 50, 100, 200, and 400 μM. Cell viability was assessed using the WST-1 assay after treatment with nicotine for 24 h. (b) The cells were incubated for 24 h with different nicotine concentrations, namely, 0, 50, 100, 200, and 400 μM. ROS generation was detected using the fluoroprobe, 2′,7′-dichlorodihydrofluorescein diacetate. *P < 0.05 or ** *P* < 0.01 compared with the controls. (c) ROS formation was detected using ROS-sensitive fluorescent dye. (d) The HK-2 cells were exposed to nicotine (200 μM for 24 h) with or without pretreatment with hexamethonium chloride (1 mM) for 3 h. **P < 0.01 compared with the controls. ††*P* < 0.01 compared with nicotine treatment. Each column represents the mean ± the standard error of the mean. The data are representative of at least three independent experiments.

The treatment of the HK-2 cells with nicotine reduced their viability in a dose-dependent manner, which was determined by the WST-1 assay (Fig 2A). The generation of ROS was detected using the ROS-sensitive fluorescent dye, DCF-DA. After nicotine treatment for 24 h, the intensity of DCF-DA fluorescence increased in a dose-dependent manner (Fig 2B and 2C). Nicotine treatment significantly increased the intensity of DCF-DA fluorescence, and this increase was moderated by inhibiting the nAChRs with hexamethonium chloride (Fig 2D).

### Effects of nicotine on the expression of mitogen-activated protein kinases and nuclear factor-κB in HK-2 cells

**Fig 3:**
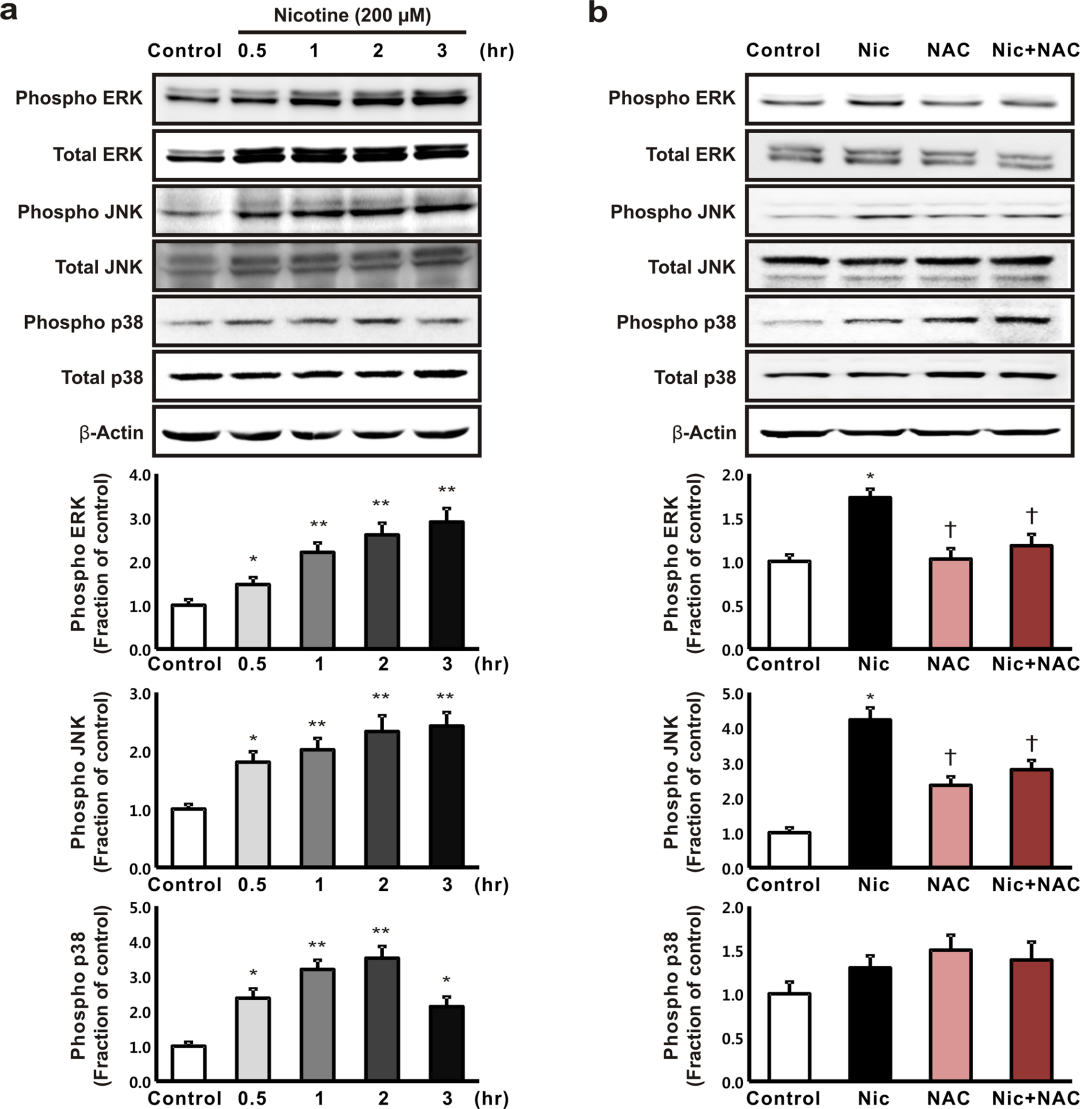
The effect of nicotine on the expression of mitogen-activated protein kinases (MAPKs) in human tubular epithelial cells. (a) The HK-2 cells were treated with 200 μM nicotine for 0.5, 1, 2, and 3 h, then the levels of expression of the phosphorylated extracellular signal-regulated kinase (ERK), c-Jun N-terminal kinase (JNK), and p38 MAPK proteins were determined. *P < 0.05 or **P < 0.01 compared with the controls. (b) The cells were exposed to nicotine (200 μM for 1 h) with or without pretreatment with 10 mM *N*-acetyl-L-cysteine for 1 h, then the levels of expression of the phosphorylated ERK, JNK, and p38 MAPK proteins were determined. *P < 0.05 compared with the controls. †*P* < 0.05 compared with nicotine treatment. Each column represents the mean ± the standard error of the mean. The data are representative of at least three independent experiments.

After the incubation of the HK-2 cells with 200 μM nicotine, the levels of expression of p-ERK, p-JNK, and p-p38 MAPK increased, and the levels of expression of the total ERK, JNK, and p38 MAPK were unaffected (Fig 3A). The nicotine-dependent increases in the expression of p-ERK and p-JNK were suppressed when the cells were pretreated with 10 mM NAC for 1 h, but the expression of p-p38 MAPK was not affected by NAC pretreatment (Fig 3B).

**Fig 4:**
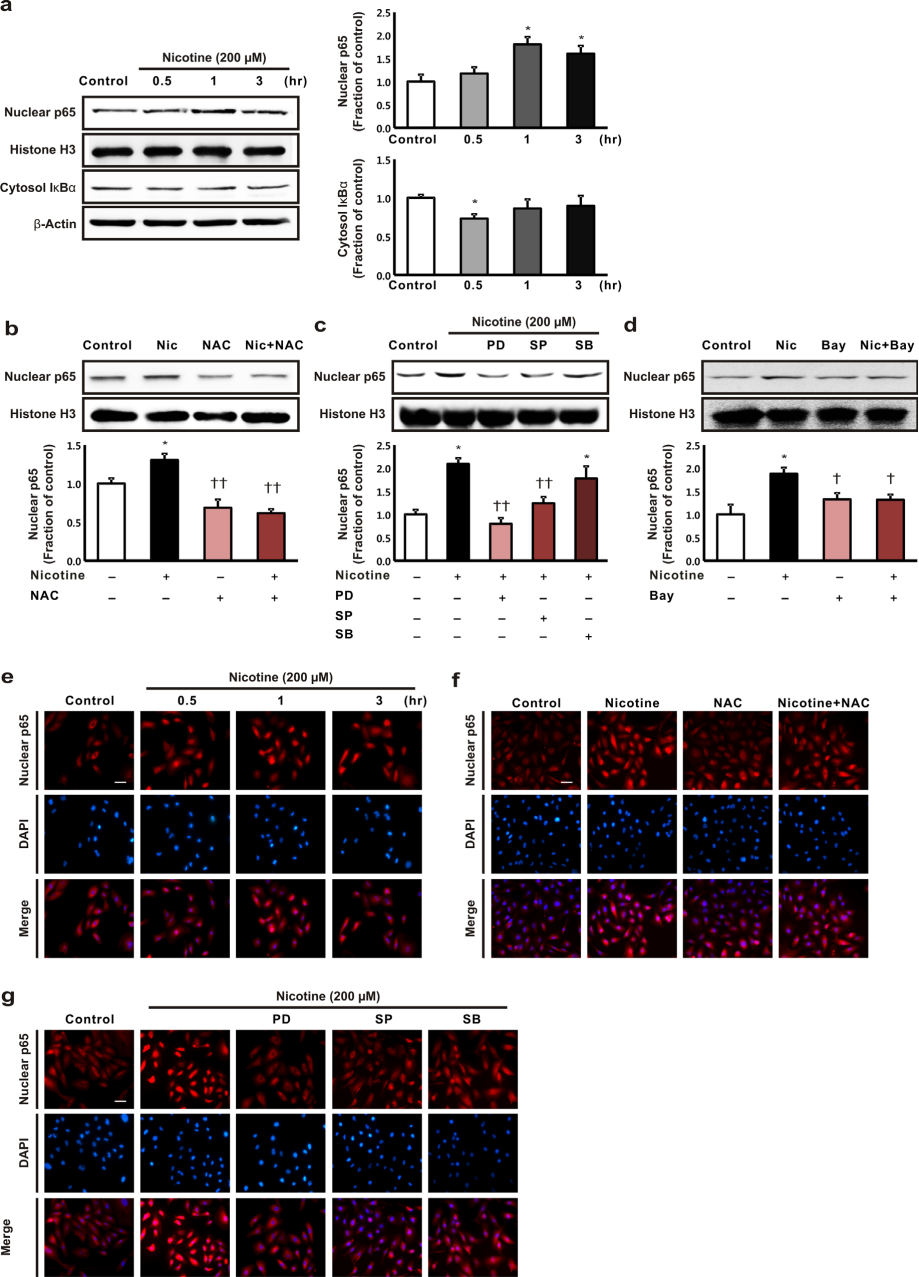
The effect of nicotine on the expression of nuclear factor-κB (NF-κB) in human tubular epithelial cells. (a) The HK-2 cells were exposed to nicotine (200 μM) for 0.5, 1, and 3 h, then the levels of the expression of the NF-κB p65 subunit and the cytosol nuclear factor of kappa light polypeptide gene enhancer in B-cells inhibitor, alpha (IκBα) proteins were determined. *P < 0.05 compared with the controls. (b) The cells were exposed to nicotine (200 μM for 1 h) with or without pretreatment with 10 mM *N*-acetyl-L-cysteine for 1 h. (c) The cells were incubated with nicotine (200 μM for 1 h) after pretreatment for 1 h with PD98059, an extracellular signal-regulated kinase inhibitor, SP600125, a c-Jun N-terminal kinase inhibitor, or SB203580, or a p38 mitogen-activated protein kinases inhibitor. (d) The cells were exposed to nicotine (200 μM for 1 h) with or without pretreatment for 1 h with Bay 11–7082, an NF-B inhibitor. *P < 0.05 compared with the controls. †*P* < 0.05 or ††*P* < 0.01 compared with nicotine treatment. Each column represents the mean ± the standard error of the mean. The data are representative of at least three independent experiments. (e, f, and g) Immunofluorescence of the NF-κB p65 subunit. Original magnification, 200 ×. Scale bar = 50 μm.

We investigated the effects of nicotine on the expression of the NF-κB p65 subunit in nuclear extracts and on the expression of total nuclear factor of kappa light polypeptide gene enhancer in B-cells inhibitor, alpha (IκBα) within the cytosol from HK-2 cells. After the incubation of the HK-2 cells with nicotine for 1 h, the cells exhibited an increase in the expression of the NF-κB p65 subunit compared with the control cells. The cytoplasmic expression of total IκBα began to decrease after 0.5 h and then it returned to the levels observed before treatment (Fig 4A). NAC pretreatment of the HK-2 cells reversed the nicotine-induced increase in the expression of the NF-κB p65 subunit (Fig 4B). We investigated the effects of specific inhibitors, including ERK (PD98059), JNK (SP600125), and p38 (SB203580) MAPK inhibitors, on the expression of the NF-κB p65 subunit to determine whether the MAPK pathway modulates NF-κB pathway signaling in nicotine-treated HK-2 cells. The ERK and JNK inhibitors attenuated the nicotine-induced increase in NF-κB p65 subunit expression, but the expression of the NF-κB p65 subunit was not affected by the p38 MAPK inhibitor (Fig 4C). The nicotine-induced increase in the expression of the NF-κB p65 subunit was also attenuated by Bay 11–7082, an NF-κB inhibitor (Fig 4D). Immunofluorescence analysis confirmed that nicotine treatment increased the nuclear translocation of the NF-κB p65 subunit and that this decreased following treatment with NAC, and the ERK and JNK inhibitors, but not after treatment with the p38 MAPK inhibitor (Fig 4E–4G).

### Effects of nicotine on cell cycle arrest in HK-2 cells

**Fig 5:**
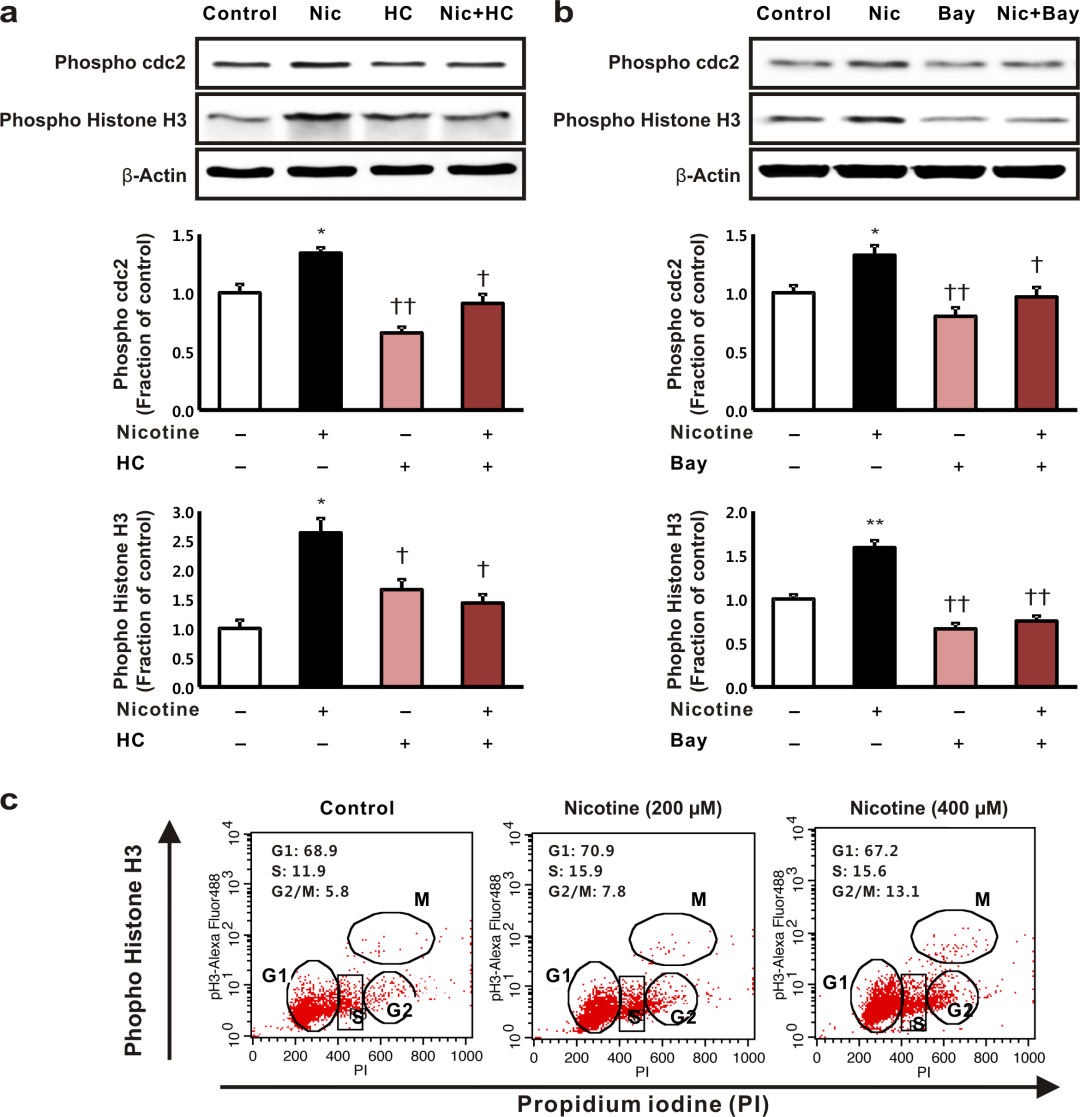
The effect of nicotine on cell cycle arrest in human tubular epithelial cells. The HK-2 cells were exposed to nicotine (200 μM for 16 h) with or without pretreatment with (a) hexamethonium chloride (1 mM) or (b) Bay 11–7082 for 3 h, then the levels of expression of the proteins cyclin B1, phosphorylated cdc2, and histone H3 were determined. *P < 0.05 or **P < 0.01 compared with the controls. †*P* < 0.05 or ††*P* < 0.01 compared with nicotine treatment. Each column represents the mean ± the standard error of the mean. The data are representative of at least three independent experiments. (c) To elucidate the distribution of the cell cycle phases, cell cycle analysis was performed using flow cytometry. The HK-2 cells were treated with nicotine (0, 200, and 400 μM for 16 h).

Tubular cell cycle arrest mediates apoptosis in a variety of renal diseases. Therefore, we investigated whether nicotine influences the arrest of the cell cycle in HK-2 cells. The levels of expression of phosphorylated cdc2 (Tyr 15) and histone H3 (Ser 10), which are markers of the G2/M phase of the cell cycle, increased after treatment with nicotine, and these increases were reduced when the cells had been incubated with the non-specific nAChR blocker, hexamethonium chloride (Fig 5A). The nicotine-induced G2/M phase arrest was reversed by Bay 11–7082 treatment (Fig 5B). When we examined the distribution of the cell cycle phases among the cells using flow cytometry, we found that the proportion of the cell population that was in the G2/M phase increased in a dose-dependent manner following nicotine treatment. The percentage of cells in the G2/M phase of the cycle increased from 5.8% to 13.1% following nicotine treatment (400 μM) (Fig 5C).

### Effects of nicotine on apoptosis in HK-2 cells

**Fig 6:**
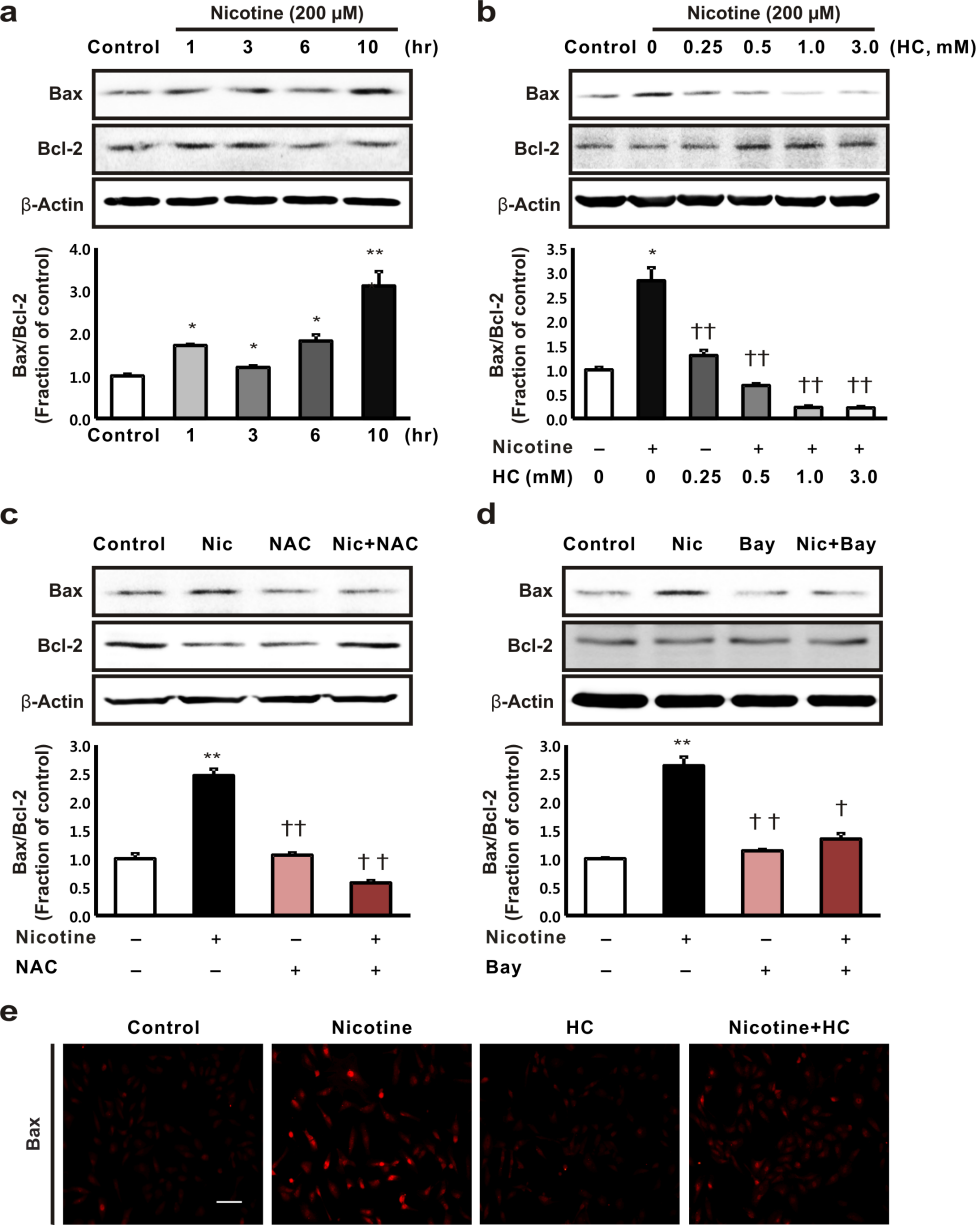
The effect of nicotine on apoptosis in human tubular epithelial cells. (a) The HK-2 cells were exposed to nicotine (200 μM) for 1, 3, 6, and 10 h, then the expression of the Bax and Bcl-2 proteins was determined. *P < 0.05 or **P < 0.01 compared with the controls. (b) The cells were pretreated with hexamethonium chloride for 3 h at different concentrations, namely, 0, 0.25, 0.5, 1, and 3 mM, then they were incubated with nicotine (200 μM for 10 h). (c) The cells were exposed to nicotine (200 μM for 10 h) with or without pretreatment with 10 mM *N*-acetyl-L-cysteine for 1 h. (d) The cells were exposed to nicotine (200 μM for 10 h) with or without pretreatment with Bay 11–7082, which is an NF-B inhibitor, for 1 h. **P < 0.01 compared with the controls. †*P* < 0.05 or ††*P* < 0.01 compared with nicotine treatment. Each column represents the mean ± the standard error of the mean. (e) Bax expression (red) was examined in the HK-2 cells after treatment with nicotine (200 μM for 10 h) and pretreatment with hexamethonium chloride (1 mM for 3 h). Original magnification, 200 ×. Scale bar = 50 μm. HC, hexamethonium chloride. The data are representative of at least three independent experiments.

We assessed the effects of nicotine on apoptosis in HK-2 cells. Nicotine increased Bax expression while inhibiting that of Bcl-2 in a time-dependent manner, which resulted in an increased Bax/Bcl-2 ratio in the HK-2 cells (Fig 6A). To determine whether the apoptotic effect of nicotine was mediated by the nAChRs, we analyzed the nicotine-induced apoptosis in HK-2 cells that had been pretreated with hexamethonium chloride. The higher Bax/Bcl-2 ratio was rectified in a dose-dependent manner in nicotine-treated HK-2 cells that had been pretreated with hexamethonium chloride (Fig 6B). In addition, the increase in the Bax/Bcl-2 ratio was attenuated by NAC and Bay 11–7082 in nicotine-treated HK-2 cells (Fig 6C and 6D). The immunofluorescence analysis also confirmed that hexamethonium chloride treatment reduced the expression of Bax, which increased following nicotine treatment (Fig 6E).

**Fig 7:**
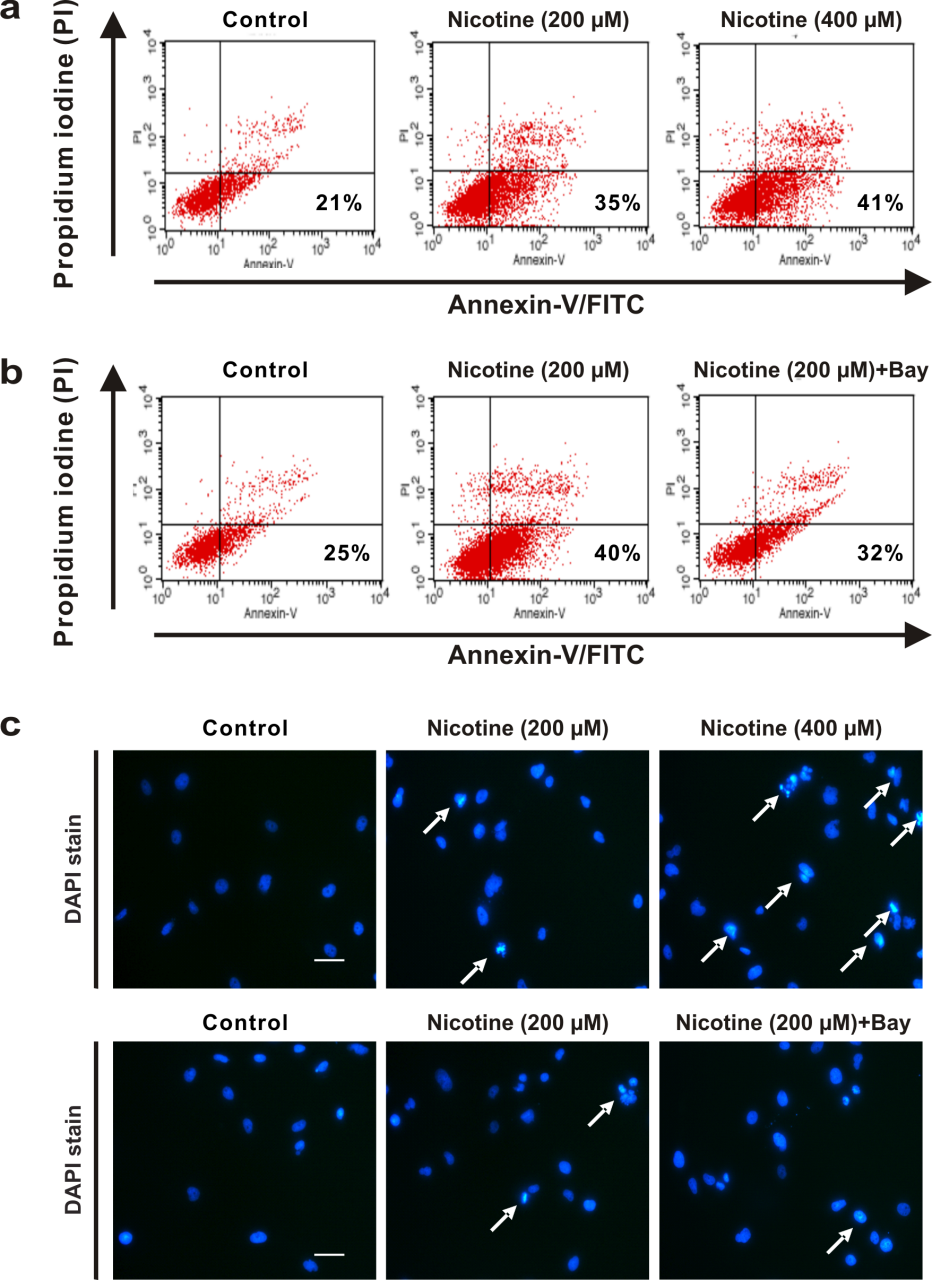
The annexin V/propidium iodide and 4′-6-diamidino-2-phenylindole staining assay. (a) The HK-2 cells were treated with nicotine at 200 or 400 μM for 24 h. (b) The cells were exposed to nicotine (200 μM) for 24 h with or without pretreatment with Bay 11–7082 for 1 h, a nuclear factor-κB inhibitor, and the percentages of the cells residing in the lower right regions of the scatter plots of annexin V-fluorescein isothiocyanate staining, which represented the apoptotic cells, were determined. (c) Chromatin condensation and apoptotic bodies were stained bright blue (arrow) in the HK-2 cells that had been treated with nicotine (200 or 400 μM for 24 h), with or without pretreatment with Bay 11–7082. The nuclear morphologies were examined using fluorescence microscopy. Original magnification, 200 ×. Scale bar = 50 μm.

We confirmed the apoptotic effect of nicotine on HK-2 cells using annexin-V binding and PI staining. HK-2 cells treated with 200 μM or 400 μM nicotine for 24 h showed a significant progressive increase with respect to annexin V-positive/PI-negative staining compared with the control cells. Pretreatment with Bay 11–7082 reduced the numbers of apoptotic cells (Fig 7A and 7B). We also detected bright blue apoptotic nuclei, which contained condensed chromatin, and apoptotic bodies in HK-2 cells that had been treated with nicotine; these effects were attenuated when the cells were pretreated with Bay 11–7082 (Fig 7C).

## Discussion

This study investigated the effect of nicotine on and it explored the potential mechanisms underlying apoptosis in HK-2 cells. The main finding from this study is that nicotine-induced oxidative stress enhanced the phosphorylation of the ERK and JNK signaling pathways, which resulted in the nuclear translocation of the NF-κB p65 subunit in HK-2 cells. Moreover, nicotine promoted the expression of apoptotic markers, including the Bax/Bcl-2 ratio, and it increased annexin V binding by activating the NF-κB signaling pathway. In addition, cell cycle arrest at the G2/M phase might be involved in nicotine-induced apoptosis in HK-2 cells.

Although the findings from several studies have demonstrated that nicotine causes apoptosis by inducing oxidative stress or p53 and p21 expression in neuronal and non-neuronal cells [26, 27], nicotine, which is a major component of cigarette smoke, promotes angiogenesis and it inhibits apoptosis in cancer cell lines, thereby aiding cancer progression [28, 29]. These discrepancies in relation to the pathophysiologic role of nicotine might be associated with differences in the models, and in the analysis and the interpretation of the results [10].

An imbalance between cell survival and cell death, which is a key feature of many degenerative and inflammatory diseases, may be caused by an aberrant turnover in ROS. Nicotine induces ROS generation in a variety of tissues, and it contributes a major proportion of the net oxidative stress imposed by cigarette smoking [26, 30, 31]. Our results also indicate that nicotine treatment of HK-2 cells causes a dose-dependent increase in ROS generation. Notably, nicotine increases oxidative stress, which causes the progression of diabetic nephropathy and chronic kidney disease in *in vivo* experimental models [6, 32]. Consistent with these observations, our *in vitro* experiments demonstrated that the increase in oxidative stress caused by nicotine treatment may promote tubular apoptosis.

It has been reported that an increase in oxidative stress stimulates extracellular MAPK pathways [33]. Extracellular MAPK signals activate the IκB kinase complex, which regulates the transcription factor NF-κB. NF-κB is released from IκBα, and it is then translocated to the nucleus where it promotes the transcription of a large number of proteins involved in inflammation, apoptosis, and cell proliferation [34]. Indeed, our findings showed that nicotine treatment increases the phosphorylation of ERK, JNK, and p38 MAPK in HK-2 cells, suggesting that nicotine activates all three MAPKs. Moreover, the nuclear expression of the NF-κB p65 subunit increased following nicotine treatment. The inhibition of ERK and JNK attenuated the nuclear expression of the NF-κB p65 subunit, while p38 MAPK inhibition did not have this effect. These findings indicate that nicotine can mediate NF-κB signaling by regulating the ERK and JNK pathways. In addition, our findings showed that NAC attenuated the activation of the ERK and JNK pathways without affecting p38 MAPK. Taken together, these results suggest that the ERK and JNK signaling pathways may modulate the activation of NF-κB signaling by inducing oxidative stress in nicotine-treated HK-2 cells.

The apoptosis pathway is regulated by members of the Bcl-2 protein family, which regulate the changes in the permeability of the mitochondrial outer membrane. Bax, a member of the Bcl-2 protein family, initiates apoptosis when it binds to the mitochondrial outer membrane, thereby changing its permeability and releasing apoptotic proteins [35]. The balance between pro-apoptotic Bax and the anti-apoptotic Bcl-2 proteins plays a major role in initiating the apoptotic pathway. The findings from our study demonstrated that nicotine increased the expression of Bax and reduced the expression of Bcl-2, which increased the Bax/Bcl-2 ratio in a dose-dependent manner. Alterations in the levels of expression of these apoptosis-related proteins may underlie nicotine-induced apoptosis in HK-2 cells.

Data that describe the physiologic roles of the nAChRs are limited. However, nAChRs are expressed within the nervous system and at the neuromuscular junctions, and they are important targets for pharmaceutical drug development in human beings [36]. Therefore, the nAChRs that are located on the postganglionic sympathetic nerve terminals could influence renal hemodynamics. Meanwhile, evidence is accumulating that suggests that non-neuronal cells express nAChRs [11–13]. The expression of nAChRs by non-neuronal cells indicates that these receptors possess functions that are independent of neurotransmission. Although the physiologic roles of the nAChRs in the kidney have not been clearly defined to date, recent epidemiologic studies have demonstrated that multiple genetic variants in the nAChR gene family are associated with insulin resistance, kidney function, and albuminuria that are not associated with cigarette smoking [37, 38]. Our results demonstrated that HK-2 cells, which are proximal tubular epithelial cells, also express nAChR subunits. Furthermore, hexamethonium chloride, which is a non-specific nAChR blocker, counteracted the effect of nicotine on the increase in the Bax/Bcl-2 ratio, which indicates that nAChRs are functionally involved in nicotine-induced apoptosis in HK-2 cells.

Apoptosis is a physiological form of programmed cell death that increases in renal and non-renal diseases, and it allows organisms to dispose of unwanted or defective cells [39]. In this respect, the modulation of the cell cycle at the G1/S or G2/M phases has been associated with programmed cell death [15]. Our results indicate that nicotine treatment induces the arrest of the cell cycle at the G2/M phase in HK-2 cells, which was demonstrated by the increased levels of expression of phosphorylated cdc2 and histone H3 and by the increases in the numbers of cells in the G2/M phase that were detected using flow cytometry, and that this may contribute to the progression of apoptosis. However, blocking the nAChRs with hexamethonium chloride or inhibiting NF-κB signaling reduced the level of arrest at the G2/M phase, which enabled the cells to progress through G2 and into the M phase. The nicotine-induced arrest of the cell cycle may affect an intrinsic apoptosis pathway via the NF-κB signaling pathway. Therefore, the nicotine-induced arrest of the cell cycle at the G2/M phase may play an important role in the apoptosis of HK-2 cells. Further investigations are required to clarify the role of nicotine in the cell cycle arrest-mediated apoptosis of HK-2 cells.

The findings from the flow cytometric analysis of the annexin V/PI staining suggested that nicotine treatment increased the number of apoptotic cells in a dose-dependent manner compared with untreated HK-2 cells. The annexin V/PI staining method is widely used in flow cytometric studies to detect apoptosis and necrosis. Apoptotic changes induce the binding of annexin V to phosphatidylserine in the outer leaflet of the plasma membrane, while PI, a fluorescent deoxyribonucleic acid intercalator, permeates the necrotic cell membrane [40]. While the flow cytometric analysis of the annexin V/PI staining determined that the number of apoptotic cells increased after the treatment of the HK-2 cells with nicotine, the number of apoptotic cells reduced when the cells were treated with an NF-κB inhibitor. Therefore, our flow cytometric data taken together with the data from the immunoblot analysis confirmed that nicotine-induced apoptosis is mediated by the NF-κB signaling pathway in HK-2 cells.

## Conclusions

The data from the present study indicate that nicotine induces apoptosis in HK-2 cells through the nAChRs. Nicotine induced apoptosis in HK-2 cells by inducing ROS generation that activated the NF-κB signaling pathway via the MAPK pathway and cell cycle arrest at the G2/M phase. However, given that this was an *in vitro* study, it is difficult to determine whether our findings are representative of smoking in real life. Further studies are needed to determine whether nAChR-depleted mice exposed to long-term smoking are prevented from showing nicotine-induced renal injury.
